# Primary thymus tumors: retrospective case analysis at a reference center in Latin America, 2011–2019

**DOI:** 10.1186/s12885-021-07920-7

**Published:** 2021-03-16

**Authors:** Diego F. Scarpetta-Gonzalez, Eliana Isabel Morales, Luz Fernanda Sua, Mauricio Velásquez, Saveria Sangiovanni, Liliana Fernández-Trujillo

**Affiliations:** 1 Department of Internal Medicine, Faculty of Health Sciences, Universidad Icesi, Calle 18 # 122-135, Cali, 7600032 Colombia; 2 Department of Internal Medicine, Pulmonology Service, Fundación Valle del Lili, Carrera 98 # 18-49, Cali, 7600032 Colombia; 3 Faculty of Health Sciences, Universidad Icesi, Calle 18 # 122-135, Cali, 7600032 Colombia; 4 Department of Pathology and Laboratory Medicine, Fundación Valle del Lili, Carrera 98 # 18-49, Cali, 7600032 Colombia; 5 Department of Surgery, Thoracic Surgery Service, Fundación Valle del Lili, Carrera 98 # 18-49, Cali, 7600032 Colombia; 6 Clinical Research Center, Fundación Valle del Lili, Carrera 98 # 18-49, Cali, 7600032 Colombia; 7 Department of Internal Medicine, Pulmonology Service, Interventional Pulmonology, Fundación Valle del Lili, Avenida Simón Bolívar. Carrera 98 # 18-49, Tower 6, 4th Floor, 7600032 Cali, Colombia

**Keywords:** Thymic neoplasm, Thymoma, Thymic carcinoma, Video-assisted thoracoscopy, Radical thymectomy

## Abstract

**Background:**

Thymic tumors are unusual neoplasms, representing 0.2 to 1.5% of tumors in humans, but correspond to 20% of mediastinal tumors and 50% of those that occur in the anterior mediastinum. They tend to appear around the fourth and fifth decades of life without gender predilection. Up to 30% of patients are asymptomatic, therefore many are incidentally diagnosed. Radical thymectomy is the treatment of choice with high survival rates when detected in the early stages.

**Methods:**

This was a retrospective descriptive study, including 18 adult patients’ diagnosis of thymic neoplasm, who were managed with surgical resection from 2011 to 2019. Information about demographics, clinical characteristics, imaging findings, surgical and medical management, plus histological findings was obtained and reported.

**Results:**

18 patients with thymic tumors were included, of which specific histologic studies reveled thymomas, carcinomas, neuroendocrine tumors, thymolipoma and thymic cyst. Mean age was 52.7 years, with a predominance of male population. The main symptom was dyspnea, followed by cough and chest pain. Paraneoplastic syndromes such as myasthenia gravis, aplastic anemia and Cushing syndrome were reported. 89% of cases were treated by radical thymectomy alone, while only 2 cases required chemotherapy and radiotherapy. There were no surgical complications. Mean hospital stay length was 11. 9 days, with only 1 mortality during hospital admission. 5-year survival rate was 81%.

**Conclusions:**

The treatment of choice is radical thymectomy, which has been shown to positively impact patient mortality. Early detection is key to improve patient outcomes.

## Background

Thymus tumors include neoplasms that arise from or differ from thymic cell constituents, comprising thymic epithelial tumors (thymomas, thymic carcinomas, neuroendocrine tumors), germ cell, lymphoid and hematopoietic, and mesenchymal tumors [1].

They are unusual neoplasms, representing 0.2 to 1.5% of tumors in humans, with an incidence of 0.1 to 0.5 cases per 100,000 individuals per year in the United States [2]. However, thymus tumors correspond to 20% of mediastinal tumors and 50% of those that occur in the anterior mediastinum [3]. They tend to appear around the fourth and fifth decades of life without gender predilection [4]. Approximately 25 to 30% of patients are asymptomatic, and are incidentally diagnosed, 50% being revealed by chest computed tomography (CT) [5]. When symptomatic, 40% of patients present with local symptoms triggered by mass effect (mainly chest pain, cough, snoring and dyspnea), 30% report constitutional symptoms (weight loss, fever and night sweats), and 30–50% present with paraneoplastic syndromes [4, 6].

When the tumor is found by imaging modalities, of which contrast enhanced chest CT scan is the image of choice, the next steps are to characterize and stage the lesion, stablishing the degree of local invasion, the presence of lymphadenopathy and distant dissemination. Hence allowing the identification of patients who benefit from surgical management as compared to those who require other types of interventions [7]. In fact, the National Comprehensive Cancer Network (NCCN) guidelines classifies thymic tumors into three clinical categories: localized and resectable, locally advanced and unresectable, and metastatic; a combination of surgical protocols, radiotherapy and/or chemotherapy is warranted for the last two groups, while surgery is sufficient to treat the first category [8].

The objective of this study is to describe the demographic, clinical, radiological and histological characteristics of 18 patients with thymic tumors, who were taken to surgical tumor resection at Fundación Valle del Lili, a high complexity institution in Cali-Colombia, between 2011 and 2019.

## Methods

This was a descriptive study based on clinical records of adult patients (≥ 18 years old) who had a diagnosis of thymic neoplasm and were treated at our institution from 2011 to 2019. Information about demographics, clinical characteristics, imaging findings, surgical and medical management, plus histological findings was obtained. Patients were followed up to December 2019 and the survival curve was estimated using the Kaplan-Meier method. This study was approved by the ethics committee of our institution and was developed according to the Helsinki Declaration of 1964.

## Results

### Demographic and clinical characteristics

18 patients with diagnosis of thymic tumor were treated at our institution between 2011 and 2019; 11 male (61%) and 7 females (39%), with a mean age of 52.7 years (±16.6). 3 patients were asymptomatic (16.6%). The remaining patients presented with constitutional symptoms (61.1%) of which the most common was weight loss, or local symptoms (55.5%) being the most frequent dyspnea, cough and chest pain in decreasing order. Paraneoplastic syndromes presented in 27.7% of patients, of which 2 had myasthenia gravis, 1 had a non-myastheniform neuromuscular disorder, 1 had red blood cell (RBC) aplasia and 1 had Cushing syndrome (Table 1).

**Table 1:** Baseline clinical characteristics of patients

Demographics	(***N*** = 18)
Male sex (%)	11 (61%)
Age – yr^a^	52.7 (±16.6)
**Clinical characteristics**	
**Asymptomatic patients (%)**	3 (16,6%)
**Constitutional symptoms (%)**	11 (61,1%)
Weight loss	4 (22,2%)
Fever	1 (5,5%)
Night sweats	0 (0%)
**Paraneoplastic syndromes (%)**	5 (27,7%)
Neuromuscular	3 (16.6%)
RBC aplasia	1 (5.5%)
Cushing syndrome	1 (5.5%)

^a^Value corresponds to mean and standard deviation (SD)

*RBC* Red blood cell

### Radiological characteristics

**Fig. 1:**
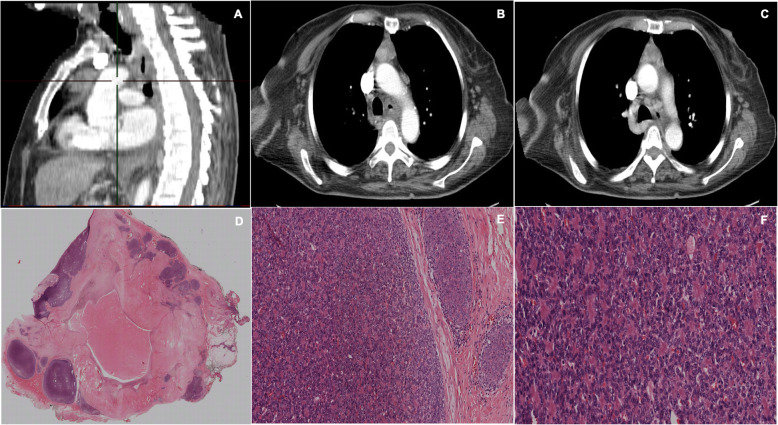
Chest CT scan. **a**, **b**, **c** coronal reconstruction. **d**, **e**, **f**. Sagittal scans. A well-defined mass of lobulated contours is observed, which compromises the anterior mediastinum towards the right hemithorax and extends in the cephalon-caudal direction towards the cardio phrenic recess on the ipsilateral side. It is homogenous, with soft tissue densify and it enhances with contrast medium. It is 13 × 10 × 15 cm, with few calcifications, and in close contact with the ascending aorta. There is a fatty plane between the superior vena cava and the mass

Chest imaging showed that 14 cases (77.7%) involved the anterior mediastinum, 2 cases (11%) compromised the left parahiliar region, 1 case (5.5%) the middle mediastinum and 1 case (5.5%) the left prevascular space (Fig. 1). The longest side of the tumors had a mean of 7.5 cm (± 5.37). Tumor density was reported in 13 patients; it was heterogenous in 53.8% of patients with calcifications in 30.8%. Only one patient presented with pleural effusion.

Most patients had a localized disease; using the Masaoka-Koga and TNM staging system for malignant tumors, 13 patients (76.4%) were classified as stage I for both systems, 3 cases (17.6%) stage IV and IVB respectively (1 patient had metastasis in deep intrathoracic/cervical lymph nodes and 2 had distant metastasis) and 1 patient (6.2%) was classified as stage III (MK) or stage II (TNM) cancer due to invasion of the pericardium. In one patient the information was not available (Table 2).

**Table 2:** Masaoka-Koga, TNM staging and histological diagnosis of patients

Masaoka-koga	(***N*** = 17)
age I	13 (76.4%)
Stage II	0 (0%)
Stage III	1 (6.2%)
Stage IV	3 (17.6%)
**TNM staging system**	**(N = 17)**
Stage I (T1a,b N0M0)	13 (76.4%)
Stage II (T2N0M0)	1 (6.2%)
Stage IIIA (T3N0M0)	0 (0%)
Stage IIIB (T4N0M0)	0 (0%)
Stage IVA (Any T, N1M0/ Any TN0-1M1a)	0 (0%)
Stage IVB (Any T, N2M0-M1a/ Any T, Any N, M1b)	3 (17.6%)
**Histological diagnosis**	**(N = 18)**
Thymoma	**13 (72.2%)**
Type A	4 **(**30.8%)
Type AB	2 (15.4%)
Type B1	6 (46.1%)
Type B2	1 (7.7%)
Thymic neuroendocrine tumors	2 (11.1%)
Thymic carcinoma	1 (5.5%)
Thymolipoma	1 (5.5%)
Thymic cyst	1 (5.5%)

### Surgical resection

None of the cases had a biopsy performed prior to the surgical resection, which was done by thoracoscopy in 12 cases (66.6%), sternotomy in 5 patients (27.8%) and thoracotomy was performed in 1 case (5.5%). There were no intra or postsurgical complications, particularly pneumothorax or phrenic nerve lesion. Median blood loss was retrieved for 16 patients, with a median of 75 cc (IQR 262.5). In 16 cases (89%) treatment consisted solely of surgical resection; 2 cases required additional therapy: one received chemotherapy after surgery and another required chemotherapy plus whole brain radiotherapy for brain metastasis.

### Histological findings

Histopathological study found 13 cases (72.2%) to be thymomas (type A in 30.8%, type AB 15.4%, type B1 46.1%, type B2 7.7%); 2 neuroendocrine tumors (11.1%), 1 thymic carcinoma (5.5%), 1 thymolipoma (5.5%) and 1 thymic cyst (5.5%) (Table 2), (Figs. 2, 3, 4, 5, 6, 7, 8, 9, 10).

**Fig. 2:**
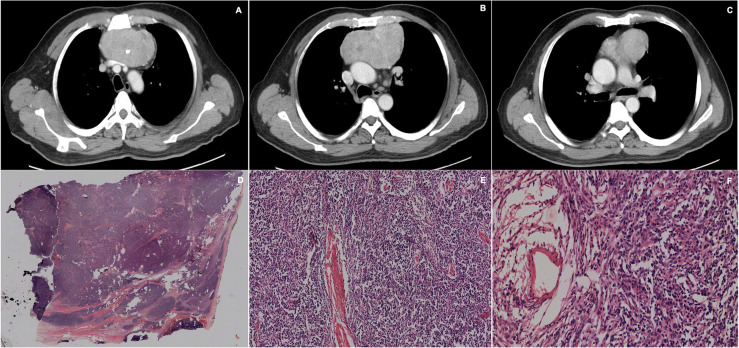
**a**, **b**, **c**. Chest CT scan showing a lobulated, well-defined contoured mass in the anterior mediastinum, which appears to be made up of multiples, smaller masses that coalesce giving a heterogeneous appearance. It has dystrophic calcifications in the center. The lesion partially compresses the left venous brachycephalic trunk. There is no compromise of the adjacent fat and had a cleavage plane with the vascular structures and soft tissues. D, E, F. H&E staining, at 2x, 10 x and 20x magnification, displaying an encapsulated mass consisting of nodules separated by fibrous septa. The cells that occupy the nodules are of two types; cohesive, large and fusiform and lymphocytes. The predominant component is epithelial tissue, forming nodules and tassels, although there are also foci rich in lymphocytes. No Hassall corpuscles are observed, cystic zones or atypia. The lesion focally crosses the capsule but is not seen to compromise fat. A minimally invasive thymoma type A of 12 cm in diameter is diagnosed

**Fig. 3:**
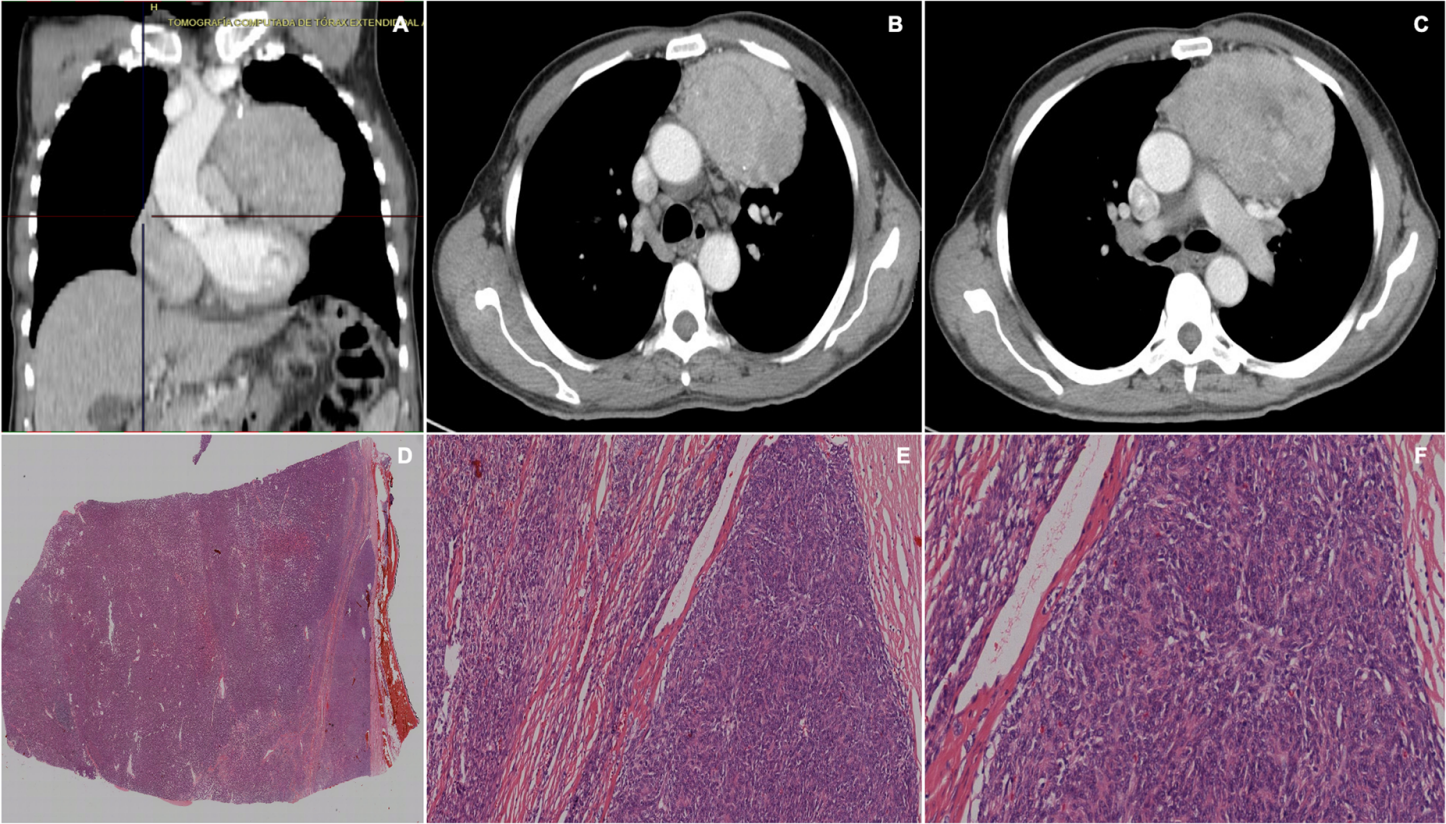
**a**, **b**, **c** Chest CT scan, coronal and sagittal scans showing a large mass of irregular contours with poorly defined edges and diffuse changes in density with hypodense areas compatible with fat or necrosis, and others with calcifications. The mass is 14.2 × 10.6 × 9 cm, located in the prevascular space, descending through the left para-aortic region towards the pulmonary aortic window. It compresses the pulmonary veins, displaces the aorta to the left and the trachea towards the right but does not alter its caliber. **d**, **e**, **f** H&E staining, evaluated at 4x, 10x and 20 x, showing very dense and lobulated proliferation of lymphocytes. The lobes are variable in size and separated by fibrous septa. There are Hassall corpuscles and also light areas that remind the medullary areas of the thymus. Atypical areas are not observed. A thymoma type A is diagnosed

**Fig. 4:**
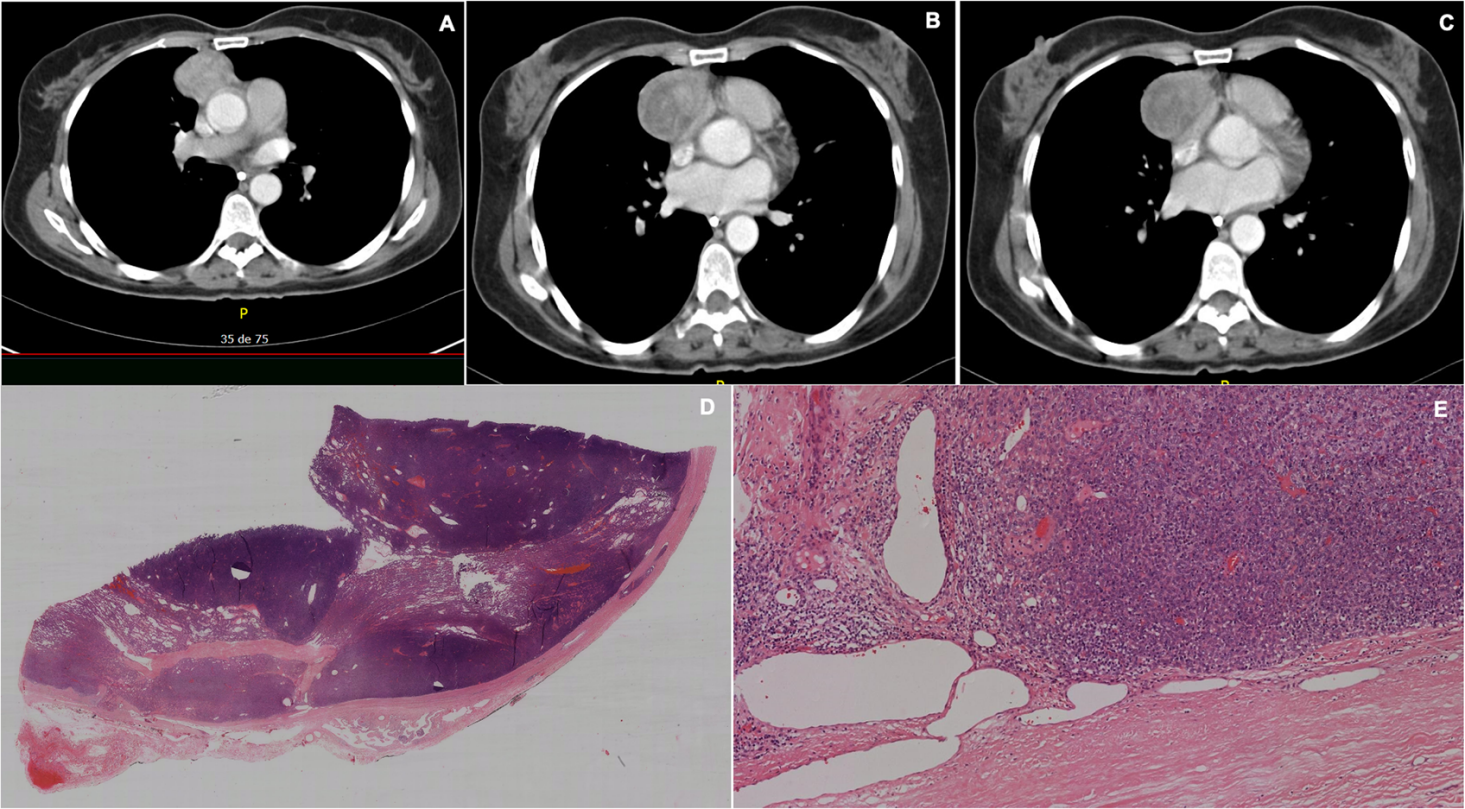
**a**, **b**, **c** Chest CT scan showing a mass of 5 × 4.5 × 5.4 cm, heterogeneous, located in the anterior mediastinum that produces posterior displacement of the right atrium, with areas than enhance with contrast and others with a necrotic aspect. **d**, **e** h&e staining, magnification at 2x and 10x, showing a lesion consisting of a mixture of 2 populations: large cohesive cells with vesicular nuclei and small and uniform cells, lymphoid in appearance, with areas that resemble the normal functioning thymus and others similar to the medullary region. Large cells are oval with a pale rounded nucleus and small nucleolus and are inconspicuously distributed among a population of lymphocytes. The lesions form large nodules separated by thick fibrous septa and is surrounded by a collagen capsule. The lesion does not exceed the capsule. Diagnosis of type B1 thymoma

**Fig. 5:**
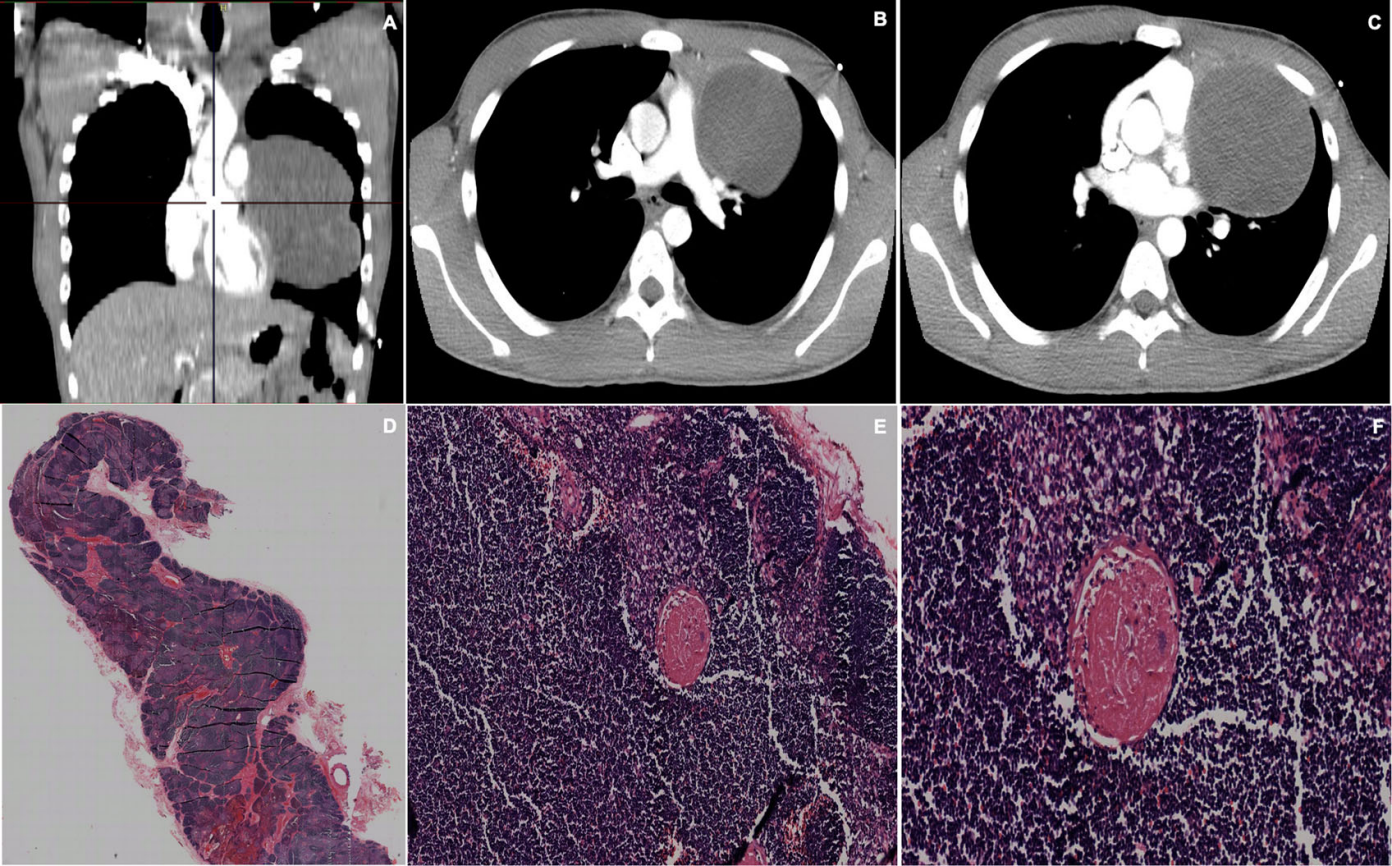
**a**, **b**, **c** Chest CT scan were a lesion of heterogenous density with predominance of soft tissue is observed. There is a liquid component inside, multilobulated septa, with well-defined observed. It is enhanced with the contrast medium, rejects the pericardium deforming it and compresses the left source bronchus and contacts the chest wall. The lesion is 13 × 9 × 8.5 cm. **d**, **e**, **f** H&E staining, magnification at 4x, 10x, and 20x showing proliferation of small, dense lymphocytes, organized in lobes, Hassall’s corpuscles and clear areas reminiscent of the thymic medullary area consistent with a cortical thymoma type B1

**Fig. 6:**
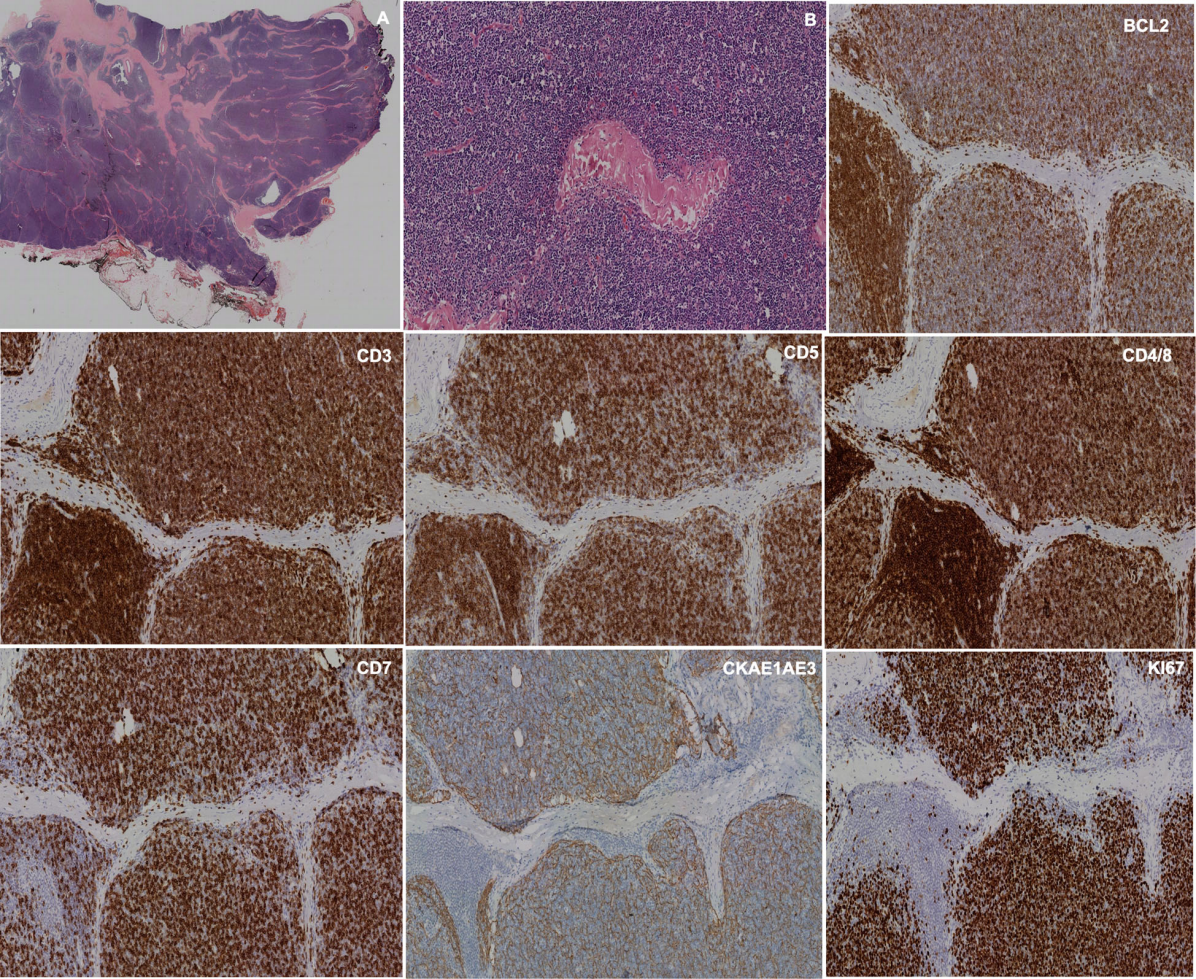
**a**, **b** H&E staining, with 10 x and 20x magnification. A pseudoencapsulated thymic lesion with fibrous septa and lymphocytic cellularity is observed. It is small in size, with conspicuous nucleolus and mitotic activity adopting a diffuse distribution pattern. With immunehistochemistry, expression is observed for T-line lymphocytes with TdT, BCL2, CD3, CD5, CD4/8 and CD7. (No representation of B line with CD20). No expression for CD15, CD30, CD117, CD56 or CD57. The epithelial weave is clearly observed with CKAE1/AE3. The rate of cell proliferation valued by Ki-67 is 80% with 5 mitoses per field of high power when studied with pHH3. It was diagnosed as a type B1 thymoma

**Fig. 7:**
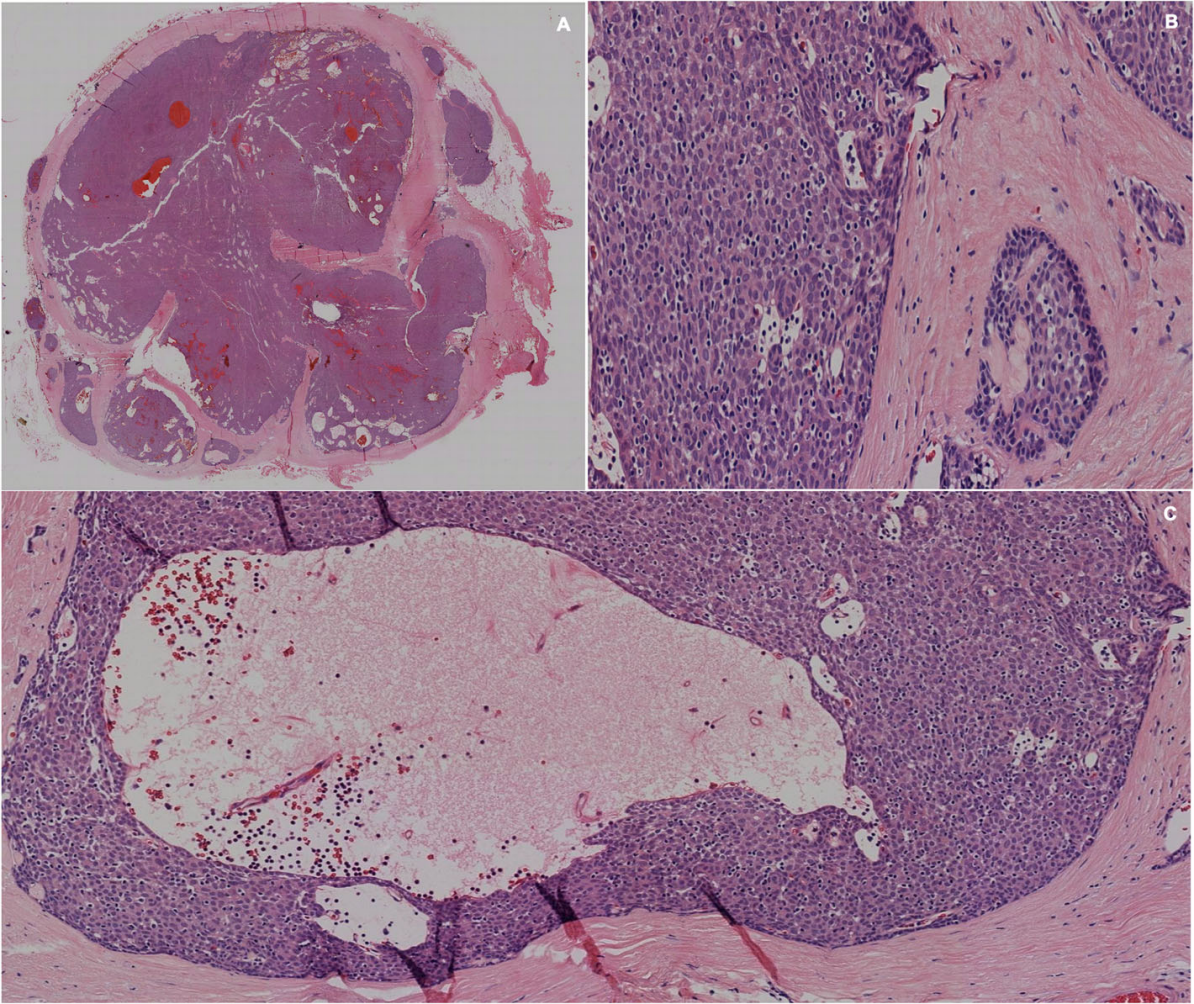
**a**, **b**, **c** H&E staining evaluated at 4x, 10x and 20x, corresponding to a delimited, capped lesion of thymic origin, composed of cells with squamous differentiation. There are no Hassall corpuscles. The lesion consists of large epithelial cells with presence of lymphocytes between them. Diagnosis of thymoma with squamous differentiation type B1/B2 is made

**Fig. 8:**
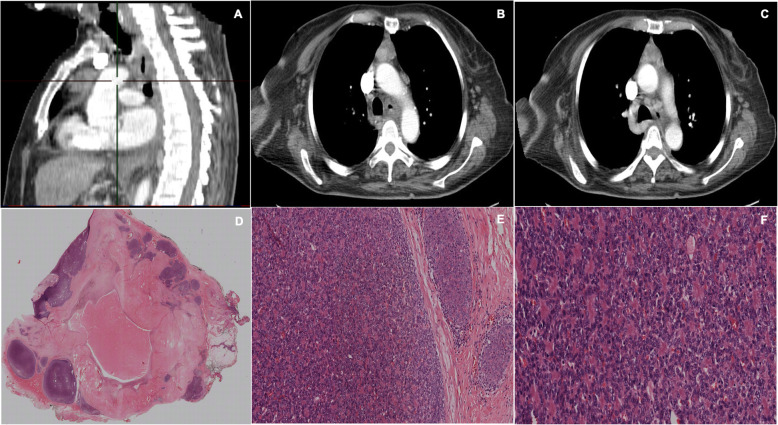
**a**, **b**, **c** Chest CT scan, 1 coronal and 2 sagittal cuts, showing a small mass localized in the anterior mediastinum with well-defined borders, heterogeneous density, without invading o compressing structures. D, E, F. Hematoxylin and eosin (H&E) staining, it is observed at 2x, 10x and 20x showing uniform cells, with little pleomorphism, cytoplasm in moderate quantity that form large solid nodules, separated by dense fibrous tissue. In some of these nodules, the tumor cells form well-differentiated rosettes. Diagnosis of an atypical carcinoid tumor originating in the thymus

**Fig. 9:**
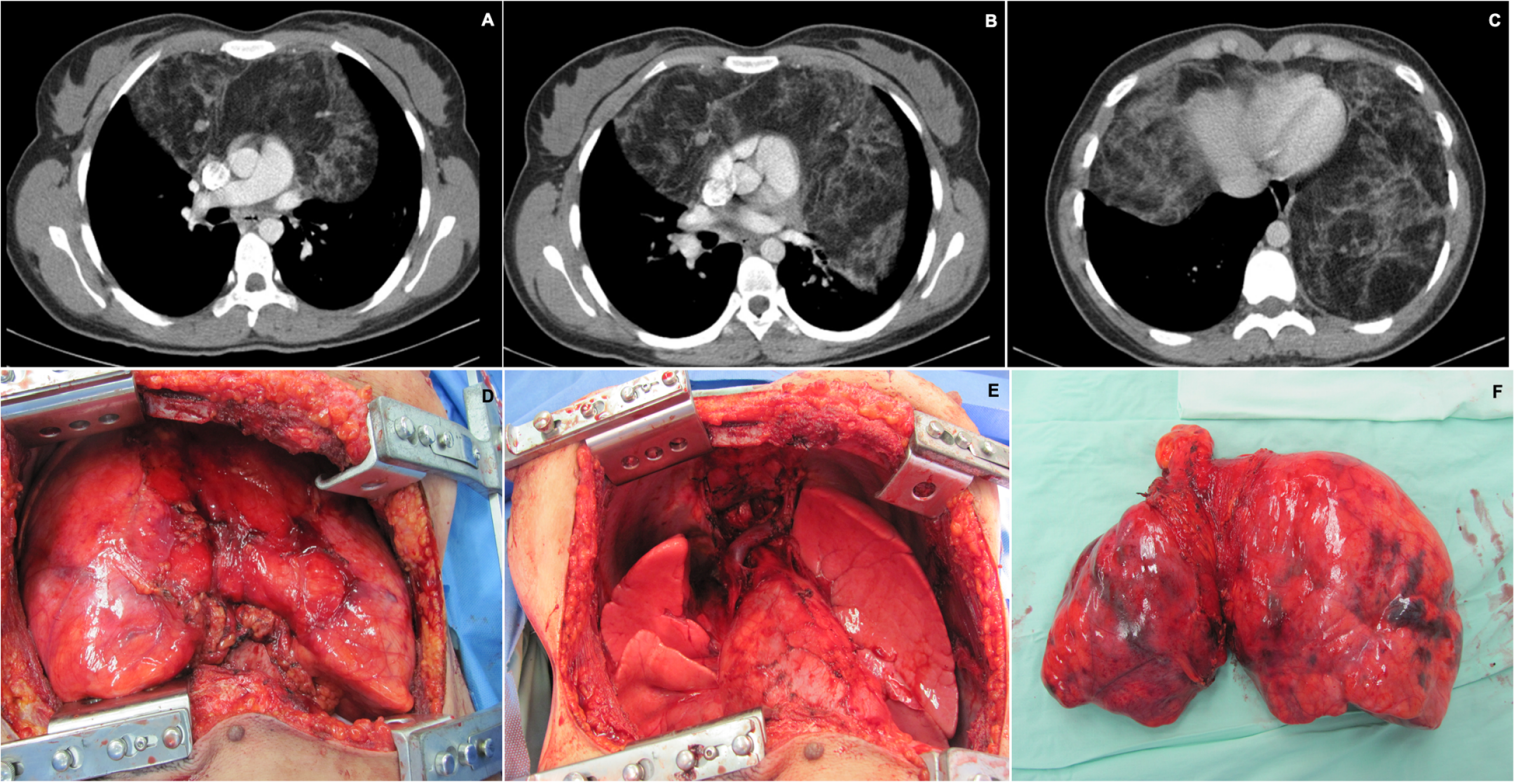
**a**, **b**, **c** Chest CT scan showing a 15 × 17 × 21 cm mass in the anterior mediastinum, with heterogeneous density. The fat content of well-defined edges that surrounds the cardio mediastinum predominates without infiltrating it and extends to the left and right thoracoabdominal area. **d** Appearance of the large tumor in the mediastinum, smooth, bilobed prior to enucleation. E. The heart, lungs and large vessels are observed after resecting the tumor lesion that had no infiltration to neighboring structures. **e** Macroscopic aspect of the resected lesion that had a histological diagnosis of thymolipoma

**Fig. 10:**
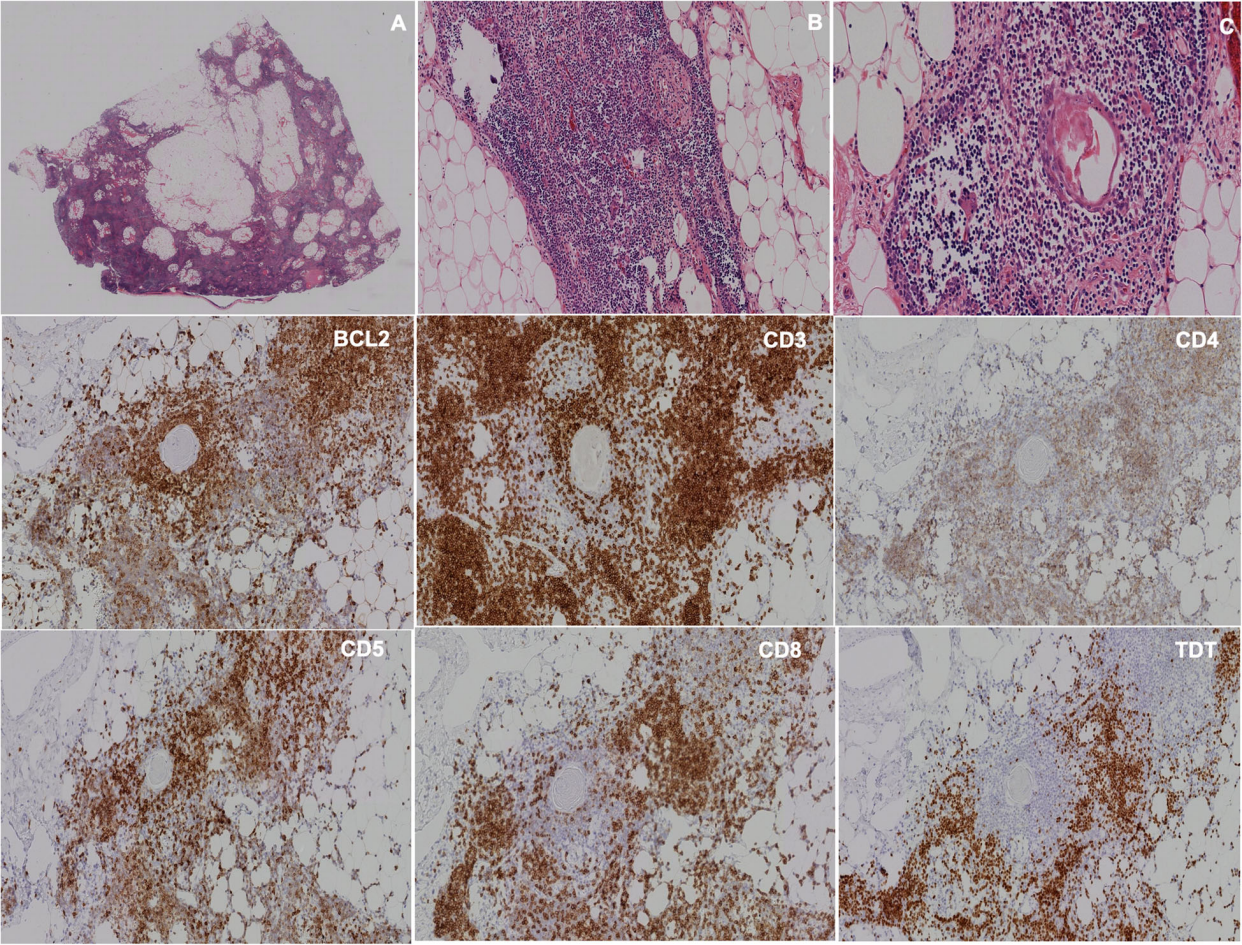
**a**, **b**, **c** H&E staining, magnification of 4x, 10x and 20x, showing an encapsulated mesenchymal lesion were the mature adipose tissue predominates. There is thymic tissue, composed by cortex and medulla with Hassall corpuscles. With immunohistochemistry, there is expression in thymic lymphocytes for CD3, CD4, CD5, CD8, TdT and BCL2 (there is no expression for CD15 or CD20). It was diagnosed as a capped thymolipoma

### Clinical outcomes

**Fig. 11:**
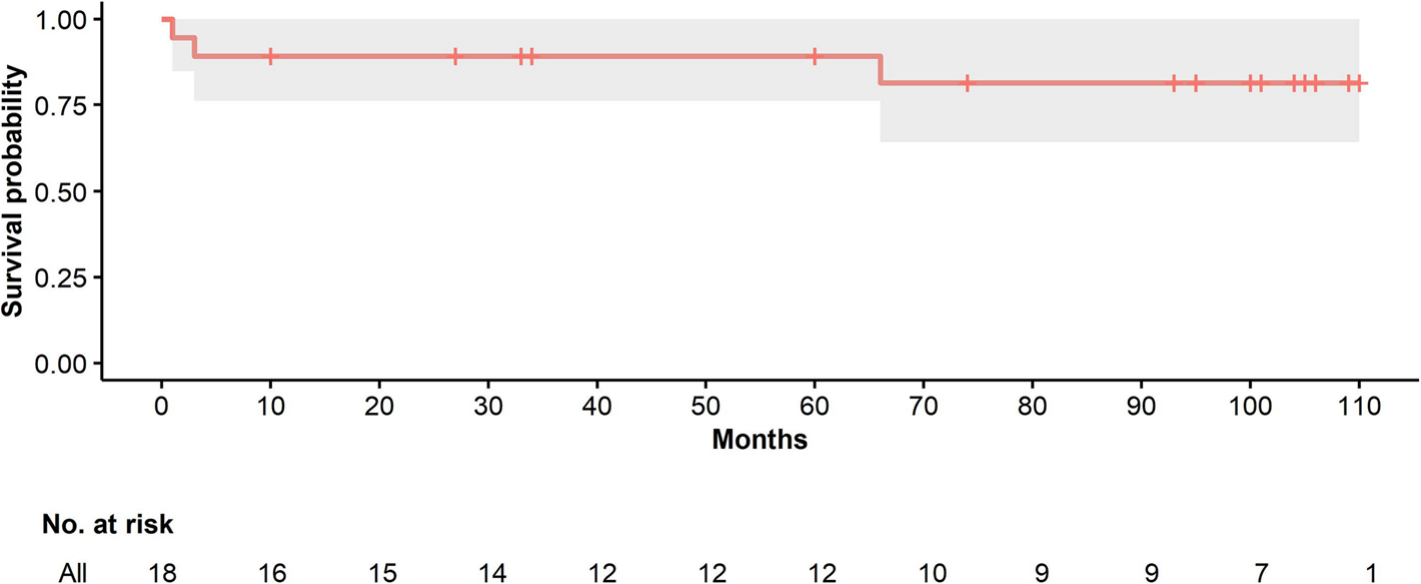
Kaplan-Meier survival estimate in patients with thymic tumors. Kaplan Meier curve showing the 5 year survival rate for these patients

Mean hospital admission length was 11.9 days; 3.3 days corresponded to intensive care unit (ICU) admission. There was only one mortality during hospitalization. After 1 month of surgery, 12 patients (66.6%) reported a significant clinical improvement during the follow up visit with the thoracic surgeon. The remaining 6 patients (33.3%) reported no change in their symptoms. The first patient had surgery on January 2011 and the last patient on May 2019, they were all followed until December 2019. Three patients died overall, and 5 patients were lost to follow up. A Kaplan-Meier curve was performed, estimating a survival probability of 81% at 5 years (Fig. 11).

## Discussion

As reported in the literature worldwide, the frequency of thymic tumors in both Latin America and Colombia is low; in fact, only 105 patients with thymomas were included in the multicenter study CLICaP-LATimus, [9] which was carried out from 1997 to 2018 in 7 Latin American countries (Mexico, Costa Rica, Colombia, Ecuador, Brazil, Peru and Argentina). In Colombia, a recent publication by Buitrago et al. reported 31 patients with thymic tumors who were treated at the National Institute of Cancerology (INC) between 2006 and 2017 [10].

The population described in our study was predominantly masculine, inconsistent with the gender distribution reported in the CLICaP-LATimus and the INC studies, were the proportion was inverted (40% men and 60% woman) [9, 10]. However, the mean age (52.7 years) was similar to the previous studies, unlike what has been reported in international cohorts, such as the European RARECAREnet projects (2000–20,007) [11] or the Japanese publication by Kanemura et al., [12] in which patients were on average a decade older at diagnosis.

Inconsistent with previous evidence, 83.4% of patients were symptomatic at diagnosis, being dyspnea the most frequent manifestation. In this study, we found that 27.7% of patients presented with paraneoplastic syndromes, consistent with the frequency reported in the literature of 30–50% [6]. Furthermore, paraneoplastic neuromuscular syndromes (myasthenia gravis, Eaton-Lambert syndrome, myotonic dystrophy, myositis, neuromyotonic, limbic encephalitis and stiff-person syndrome) are the most common similar to our findings, but hematological (RBC aplasia and pancytopenia), endocrine, dermatological and rheumatological disorders are also prevalent [6].

Symptomatic patients are more likely to have a chest image, facilitating diagnosis. Although a contrast enhanced chest computed tomography (CT) is the image of choice (Fig. 1), [7] cardiac or chest nuclear magnetic resonance imaging (NMRI) is useful in distinguishing compression from vascular invasion, in large lesions were this could be difficult to determine by CT. NMRI also allows the evaluation of the phrenic nerves and gives additional information about the involvement of the chest wall [13–15].

The National Comprehensive Cancer Network (NCCN) guidelines divide thymic cancers into three categories, according to the possibility of surgical resection. A total thymectomy and complete excision of the tumor without further treatment is the recommendation for TNM stage I tumors. When capsular invasion is present (stage II-IV) postoperative radiotherapy should be considered. For locally advanced tumors, the possibility of resection should be carefully considered by an experienced multidisciplinary team, and multimodal treatment with chemotherapy and/or radiotherapy is warranted [16]. In our study 89% patients were managed with surgery alone, leaving no residual tumor, including 13 patients with stage I cancer, 1 patient with stage II (T2N0M0) and 1 patient with stage IVB (T3N2M0). The 2 remaining patients, who had separate pleural metastasis (T4N2M1a) and extrathoracic metastasis (T4N2M1b) affecting the brain, also underwent surgery but were offered additional oncologic treatment.

There are different surgical approaches to radical thymectomy, including cervicotomy, thoracotomy, sternotomy, video-assisted thoracoscopic surgery (VATS) (Fig. 9), and robotic assisted thoracoscopic surgery (RATS). All soft tissue should be removed in the anterior mediastinum between the phrenic nerves, which is key to control patients who present with myasthenia gravis and has been shown to positively impact mortality [17, 18]. Although the standard surgical technique is median sternotomy, since it allows a complete visualization of the mediastinum, pleural spaces, presence of capsular invasion or infiltration of near-by structures, VATS has been gaining popularity given that it is a minimally invasive procedure [17, 19]. VATS has been proved consistently to deliver best perioperative outcomes compared to open surgery, due to a reduced blood loss, shorter surgical time and hospitalization, better pain management and fewer postoperative infections. However, the oncological results of VATS are still controversial regarding the completeness of resection and recurrence rates, therefore its use is widely accepted for early stage thymomas (Masaoka-Koga I, II) [19]. Nevertheless, Ning Xu et al., published a retrospective case report of 4 patients with stage III thymic tumors with invasion of the superior vena cava (SVC), who underwent VATS plus partial SVC resection without presenting major perioperative or postoperative complications, no mortality nor recurrence after 14 months of follow-up. These findings could be replicated by experienced centers worldwide giving hope to minimally invasive strategies [20]. In our patients, thoracoscopy was the most utilized approach in 66.6% of cases. In our experience there were no intra or post operatory complications, even in patients who underwent sternotomy and thoracotomy.

Definitive diagnosis was made after surgical intervention in most cases, as in our patients, since the use of biopsy is only contemplated when there is suspicion of a differential diagnosis such as germ cell tumor or goiter [21]. The World Health Organization (WHO) described a histopathological classification for thymomas, thymic carcinomas, thymic neuroendocrine tumors, among others. Specifically for thymomas, which are the most frequent, they are classified as type A, atypical type A variant, type AB, type B1-B3, micronodular thymoma with lymphoid stroma (MNT), metaplastic thymoma and other rare thymomas such as microscopic thymoma, sclerosing thymoma and lipofibroadenoma (Table 3) [22]. In our experience, thymomas were the most common histopathological finding (72.2%), similar to the INC study which reported thymomas in 70.9% of cases [10]. Our most frequent subtype was B1 (46.1%) followed by type A (30.8%).

**Table 3:** World Health Organization (WHO) histological classification of thymomas

Thymoma subtype	Obligatory criteria	Optional criteria
Type A	Occurrence of bland, spindle shaped epithelial cells (at least focally); paucity^a^ of absence of immature (TdT+) T cells throughout the tumor	Polygonal epithelial cells CD20+ epithelial cells
Atypical type A variant	Criteria of type A thymoma; in addition: comedo-type tumor necrosis; increased mitotic count (> 4/2 mm^2^); nuclear crowding	Polygonal epithelial cells CD20+ epithelial cells
Type AB	Occurrence of bland, spindle shaped epithelial cells (at least focally); abundance of immature (TdT+) T cells focally or throughout the tumor	Polygonal epithelial cells CD20+ epithelial cells
Type B1	Thymus-like architecture and cytology: abundance of immature T cells, areas of medullary differentiation (medullary islands); paucity of polygonal or dendritic epithelia cells without clustering (< 3 contiguous epithelial cells)	Hassall’s corpuscles; perivascular spaces
Type B2	Increased numbers of single or c lustered polygonal or dendritic epithelial cells intermingled with abundant immature T cells	Medullary islands; Hassall’s corpuscles; perivascular spaces
Type B3	Sheets of polygonal slightly to moderately atypical epithelial cells; absent or rare intercellular bridges; paucity or absence of intermingled TdT+ T cells	Hassall’s corpuscles; perivascular spaces
MNT^b^	Nodules of bland spindle or oval epithelial cells surrounded by an epithelial cell free lymphoid stroma	Lymphoid follicles; monoclonal B cells and/or plasma cells (rare)
Metaplastic thymoma	Biphasic tumor composed of solid areas of epithelial cells in a background of bland-looking spindle cells; absence of immature T cells	Pleomorphism or epithelial cells; actin, keratin or EMA-positive spindle cells
Rare others^c^		

^a^Paucity versus abundance: any area of crowded immature T cells or moderate numbers of immature T cells > 10% of the investigated tumor are indicative of “abundance”

^b^MNT, micronodular thymoma with lymphoid stroma

^c^Microscopic thymoma; sclerosing thymoma; lipofibroadenoma [22]

Long-term survival of thymic tumors, particularly thymomas, tends to be favorable after radical resection. A retrospective analysis of 62 patients with thymomas who underwent thymectomy, reported an overall 5- and 10-year survival rate of 85.36 and 78.20%, respectively. In this cohort, patients < 50 years old, early Masaoka stages (I and II), histological type (type A, AB, B1) and lack of recurrence were independent prognostic factors of survival [23]. In our experience, survival rate at 5 years was 81%, similar to the study cited earlier. We had three mortalities, of which 2 had disseminated disease.

## Conclusion

Thymic tumors are unusual in the general population but represent half of tumors affecting the anterior mediastinum, of which thymomas are the most common. The treatment of choice is radical thymectomy, which has been shown to positively impact patient mortality. Although open surgery is the standard approach, VATS has been shown to be a safe and efficacious procedure and should be considered for this group of patients. While evidence suggests the majority of patients are asymptomatic, this was not our experience, facilitating early diagnosis and possibly increasing the survival probability of our patients.

## Data Availability

All data and material are available for sharing if needed with the Corresponding Author.

## Abbreviations

CT: Computed tomography
NCCN: National Comprehensive Cancer Network
RBC: Red blood cell
TNM staging: Tumor, nodule, metastasis staging
MK: Masaoka-Koga
IQR: Interquartile range
ICU: Intensive care unit
INC: National Institute of Cancerology
NMRI: Nuclear Magnetic Resonance Imaging
VATS: Video-assisted thoracoscopic surgery
WHO: World Health Organization
MNT: Micronodular thymoma with lymphoid stroma.
SVC: Superior vena cava
